# Association between the atherogenic index of plasma and the occurrence of acute kidney injury in critically ill patients with sepsis: A retrospective study

**DOI:** 10.1371/journal.pone.0337903

**Published:** 2025-12-01

**Authors:** Bing Wu, Pengli Wei, Jiaxiang Deng, Yuanyuan Rui

**Affiliations:** 1 Department of Emergency, the Second People’s Hospital of Lu’an City, Lu’an, Anhui, China; 2 Department of Emergency, the First Affiliated Hospital of Wannan Medical College, Wuhu, Anhui, China; Purdue University, UNITED STATES OF AMERICA

## Abstract

**Background:**

The atherogenic index of plasma (AIP) is a recognized marker of atherosclerosis and cardiovascular disease (CVD). However, the association between AIP and the risk of acute kidney injury (AKI) in critically ill patients with sepsis has not yet been investigated.

**Methods:**

The data used in this study were derived from the Medical Information Mart for Intensive Care (MIMIC-IV) database. The clinical outcome was the occurrence of AKI. Logistic regression was used to assess the association between AIP and the risk of AKI in sepsis patients. Restricted cubic spline (RCS) analysis was applied to explore potential non-linear relationships. Threshold analysis confirmed a turning point at this value. Subgroup analyses evaluated the consistency of the association across different strata. Mediation analysis was performed to explore potential intermediate variables.

**Results:**

Among 1,874 sepsis patients, higher AIP levels were associated with increased AKI incidence. Logistic regression showed a significant association between AIP and AKI in unadjusted and partially adjusted models, but the association was no longer significant after full adjustment. RCS analysis revealed a nonlinear relationship with a peak AKI risk at AIP = 1.333. Threshold analysis confirmed a turning point at this value. Subgroup analyses showed consistent associations, while nonlinear effects were more evident in specific groups. Mediation analysis suggested that SOFA score, creatinine, WBC count, and respiratory rate partially mediated the AIP-AKI relationship.

**Conclusion:**

AIP was nonlinearly associated with AKI in sepsis, with a clear threshold effect. This relationship was partially mediated by SOFA score, creatinine, WBC, and respiratory rate. AIP may serve as a useful marker for AKI risk assessment.

## Introduction

Sepsis is characterized by life-threatening organ dysfunction resulting from an abnormal host immune response triggered by infection [1]. Acute kidney injury (AKI) is a common complication in up to 60% of patients with critical illness and is associated with high morbidity and mortality [2]. A European multicenter study showed that 51% of patients with sepsis in the intensive care unit (ICU) developed AKI, and the mortality rate in patients with sepsis-associated AKI (SA-AKI) was 41% [3]. Although considerable progress has been made in elucidating the mechanisms behind sepsis and developing clinical treatments, the rates of occurrence and mortality have only modestly declined [4,5]. Sepsis remains a major global health issue, impacting over 30 million individuals worldwide each year [6,7]. In the U.S., the national weighted incidence of sepsis is approximately 6%, accompanied by an in-hospital death rate of 15.6% [5]. Consequently, it is essential to determine factors that increase sepsis risk in order to lower mortality.

A hallmark of type 2 diabetes mellitus (T2DM) is insulin resistance (IR), which notably elevates the risk of AKI. From a pathophysiological perspective, IR promotes inflammation, lipid abnormalities, and endothelial dysfunction, potentially exacerbating sepsis and subsequent AKI development [8]. Introduced by Dobiásová and Frohlich in 2001, the atherogenic index of plasma (AIP) – defined as the logarithmic ratio of triglycerides (TG) to high-density lipoprotein (HDL) cholesterol – is increasingly recognized as an advanced and effective indicator of dyslipidemia [9]. AIP has been shown to be closely associated with IR [10,11]. As found in the study by Bei Yin et al., there is a non-linear relationship between AIP and IR [10].

Numerous prior studies have demonstrated a clear association between the AIP and mortality [12–17], and one study has reported a significant relationship between AIP and AKI in patients with pancreatitis [18]. However, to date, no research has explored the relationship between AIP and the occurrence of AKI among critically ill patients with sepsis. Therefore, this study aimed to examine the association between AIP and AKI in patients with sepsis, thereby highlighting the potential role of insulin resistance in the pathogenesis of septic AKI.

## Methods

### Study population

**Fig 1:**
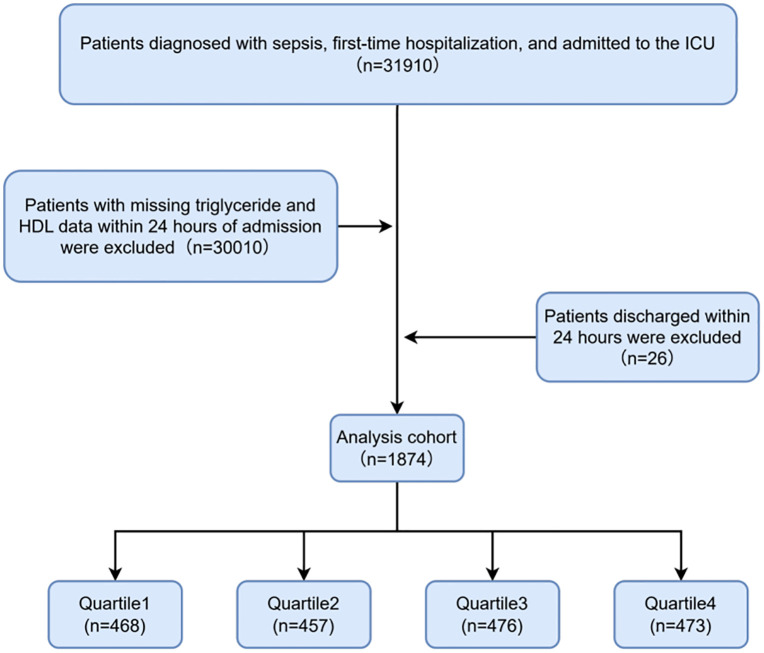
Flow chart of study participants.

This study was a retrospective analysis utilizing data from the MIMIC-IV database (version 3.0), whose full name is the Medical Information Mart for Intensive Care Database [19]. The author (Yuanyuan Rui) followed the requirements for database access and was responsible for the extraction of the data. We included patients diagnosed with sepsis who were admitted to the ICU during their first hospitalization, based on data extracted from the MIMIC-IV database. The following groups of people were excluded: (1) patients under 18 years old at the time of their initial admission (n = 0); (2) patients who discharged within 24 hours of admission (n = 26); and (3) patients lacking adequate data (triglyceride and HDL) on the first day of admission (n = 30010). Finally, 1874 participants were included in this study, which categorized them into four groups according to the quartiles of the AIP (Fig 1). Since the data used in this study were obtained from a publicly available database with all personal identifiers removed, ethical approval was not required. Ethics committee waived the requirement for informed consent.

### Data collection

The software PostgreSQL (version 13.7.2) was utilized to retrieve information. The extracted data can be divided into the following categories: Demographics, past medical history, Laboratory parameters, treatment and Severity of illness scores. The calculation of the AIP is as follows [15,20,21]:

AIP = Log (triglyceride/ HDL), with both TG and HDL-C levels are expressed in mmol/L.

All variables were obtained from the data collected within 24 hours of admission.

To prevent potential bias, variables with over 20% missing values are eliminated [22]. For those with less than 20% missing values, the “missForest” package in R Studio was used to impute the data.

### Clinical outcome

The outcome of this study was the occurrence of AKI during hospitalization.

### Statistical analysis

Continuous variables are categorized into normally distributed and nonnormally distributed data. Normally distributed data are presented as means ± standard deviations, and analysis of variance (ANOVA) is employed to assess differences between groups. In contrast, nonnormally distributed data are represented as medians with interquartile ranges (IQRs), with the Kruskal-Wallis test used for group comparisons. Categorical variables are expressed as percentages, and chi-square tests are utilized to evaluate differences between groups.

We divided patients into four groups: quartile 1 with AIP ≤ 0.44, quartile 2 with 0.44 < AIP ≤ 0.89, quartile 3 with 0.89 < AIP ≤ 1.47, quartile 4 with AIP > 1.47 [10]. First, we identified variables that were associated with outcomes via univariate logistic regression analysis. Then, we constructed three logistic regression models to examine the association between AIP and the outcomes. Model 1 was unadjusted; Model 2 adjusted for basic demographic variables; and Model 3 further adjusted for clinical covariates. The AIP was assessed using both nominal and continuous variables. The P values for trends were determined based on quartile levels.

Additionally, three restricted cubic spline (RCS) regression models were employed to explore potential nonlinear associations between AIP and clinical outcome. To further examine whether the observed associations varied across different patient subgroups, we conducted subgroup analyses, with P-values for interaction displayed in the figures. Moreover, threshold effect analysis was performed to identify potential inflection points in the relationship between AIP and the outcomes. Finally, mediation analysis was used to assess whether specific clinical variables mediated the association between AIP and the risk of adverse outcome.

A two-tailed p-value below 0.05 was deemed statistically significant. All the statistical analysis were conducted using R software (version 4.4.0) and StataMP 16.0 (Stata Corp., College Station, TX, USA).

## Results

### Baseline characteristics

A total of 1,874 patients were included in this study and categorized into four groups (Q1-Q4) based on the quartiles of AIP. Baseline characteristics of the patients across the AIP quartiles are presented in **Table 1**. Overall, 56.9% of the patients were male, with a mean age of 69.02 ± 14.99 years. As AIP increased, both the proportion of male patients and body weight showed an upward trend, whereas age tended to decrease. Regarding laboratory findings, significant differences were observed across AIP quartiles in serum sodium, creatinine, hemoglobin, blood urea nitrogen (BUN), white blood cell (WBC) count, and platelet (PLT) count (all p < 0.05). Notably, the incidence of AKI increased progressively with higher AIP levels, from 63.2% in the Q1 group to 74.6% in the Q4 group (p < 0.001).

**Table 1:** Characteristics and outcomes of patients grouped by AIP quartiles^a.^

	Total	Q1	Q2	Q3	Q4	p
AIP	1.00 ± 0.87	0.02 ± 0.33	0.67 ± 0.13	1.17 ± 0.17	2.13 ± 0.72	<0.001
Gender						<0.001
Male	1067 (56.9%)	224 (47.9%)	262 (57.3%)	292 (61.3%)	289 (61.1%)	
Female	807 (43.1%)	244 (52.1%)	195 (42.7%)	184 (38.7%)	184 (38.9%)	
Age	69.02 ± 14.99	73.74 ± 14.38	71.92 ± 13.68	68.43 ± 14.31	62.13 ± 14.90	<0.001
Race						0.001
Asian	53 (2.8%)	8 (1.7%)	17 (3.7%)	21 (4.4%)	7 (1.5%)	
Black	153 (8.2%)	49 (10.5%)	42 (9.2%)	30 (6.3%)	32 (6.8%)	
White	1091 (58.2%)	280 (59.8%)	271 (59.3%)	284 (59.7%)	256 (54.1%)	
Hispanic	47 (2.5%)	7 (1.5%)	9 (2.0%)	12 (2.5%)	19 (4.0%)	
Other	530 (28.3%)	124 (26.5%)	118 (25.8%)	129 (27.1%)	159 (33.6%)	
Weight	82.02 ± 22.80	74.76 ± 17.98	79.43 ± 20.90	85.64 ± 24.85	88.09 ± 24.31	<0.001
HR	86.20 ± 18.41	85.03 ± 18.20	85.16 ± 17.70	85.89 ± 18.71	88.66 ± 18.79	0.007
Mbp	90.80 ± 17.53	91.67 ± 17.44	92.65 ± 18.43	91.31 ± 16.30	87.65 ± 17.56	<0.001
RR	19.66 ± 5.07	19.10 ± 4.73	19.57 ± 5.07	19.62 ± 5.15	20.32 ± 5.28	0.003
SpO2	97.18(96.00, 99.00)	97.00(96.00, 99.00)	97.16(96.00, 99.00)	97.51(96.00, 99.00)	97.00(95.76, 99.00)	0.397
Sofa	1.32 (0.00, 3.00)	1.00 (0.00, 2.00)	1.34 (0.00, 2.53)	1.00 (0.00, 3.00)	2.00 (0.00, 4.00)	<0.001
Sodium	138.39 ± 5.06	137.95 ± 5.37	138.94 ± 4.30	138.71 ± 4.50	137.97 ± 5.82	0.003
Potassium	4.18 ± 0.74	4.13 ± 0.67	4.20 ± 0.78	4.18 ± 0.74	4.23 ± 0.77	0.286
Chloride	103.06 ± 5.63	103.04 ± 5.59	103.24 ± 5.10	103.24 ± 5.25	102.71 ± 6.46	0.427
Creatinine	1.39 ± 1.29	1.19 ± 1.01	1.36 ± 1.25	1.30 ± 0.99	1.73 ± 1.73	<0.001
Hemoglobin	12.03 ± 2.22	11.95 ± 2.06	12.15 ± 2.16	12.26 ± 2.16	11.75 ± 2.47	0.002
BUN	25.00 ± 18.20	22.77 ± 16.15	24.00 ± 15.74	24.28 ± 16.65	28.89 ± 22.72	<0.001
WBC	11.28 ± 5.26	10.72 ± 4.57	11.11 ± 4.91	11.30 ± 5.35	11.98 ± 6.02	0.003
PLT	217.12 ± 85.74	211.30 ± 73.73	225.40 ± 82.30	219.46 ± 86.58	212.50 ± 97.89	0.043
CHD	909 (48.5%)	216 (46.2%)	234 (51.2%)	248 (52.1%)	211 (44.6%)	0.053
Hypertension	603 (32.2%)	155 (33.1%)	159 (34.8%)	146 (30.7%)	143 (30.2%)	0.402
T2DM	412 (22.0%)	61 (13.0%)	102 (22.3%)	117 (24.6%)	132 (27.9%)	<0.001
CKD	473 (25.2%)	105 (22.4%)	119 (26.0%)	129 (27.1%)	120 (25.4%)	0.394
Vasoactive agent	612 (32.7%)	136 (29.1%)	118 (25.8%)	163 (34.2%)	195 (41.2%)	<0.001
Ventilation	1447 (77.2%)	346 (73.9%)	338 (74.0%)	371 (77.9%)	392 (82.9%)	0.002
Los_hospital	10.72(6.28, 17.82)	9.26 (5.80, 15.72)	10.88(6.75, 17.09)	10.81(5.99, 18.25)	12.27(7.13, 21.77)	<0.001
Los_icu	4.05 (1.86, 8.35)	3.96 (1.85, 6.95)	4.16 (1.89, 8.63)	3.96 (1.76, 8.10)	4.04 (1.96, 9.68)	0.193
AKI	1299 (69.3%)	296 (63.2%)	306 (67.0%)	344 (72.3%)	353 (74.6%)	<0.001

^a^AIP quartiles: Q1 (≤0.44),Q2 (0.44<AIP≤0.89),Q3 (0.89<AIP≤1.47),Q4 (SHR>1.47)

Abbreviations: AIP, atherogenic index of plasma; HR, heart rate; Mbp, mean blood pressure; RR, respiratory rate; WBC, white blood cell; PLT, platelet; BUN, blood urea nitrogen; SOFA, sequential organ failure assessment; CHD, coronary heart disease; T2DM, type 2 diabetes mellitus; CKD, chronic kidney disease; Los, length of stay; AKI, acute kidney injury

### Clinical outcomes

S1 Table presents the results of univariate logistic regression analysis, in which variables such as sex, age, race, respiratory rate, SOFA score, creatinine, WBC count, coronary heart disease (CHD), hypertension, chronic kidney disease (CKD), use of vasoactive agents, and mechanical ventilation were identified as potential confounders. **Table 2** shows the results of three logistic regression models evaluating the association between AIP and the risk of AKI. In both the unadjusted model (Model 1) and the partially adjusted model (Model 2), AIP—whether treated as a continuous variable or categorized into quartiles—was significantly associated with the occurrence of in-hospital AKI among septic patients. Patients in the higher AIP quartiles (Q3 and Q4) had a significantly increased risk of AKI. However, after further adjustment for clinical variables including respiratory rate, SOFA score, creatinine, WBC count, and comorbidities (Model 3), the association between AIP and AKI was no longer statistically significant. Only the third quartile (Q3) remained associated with a modestly elevated risk (OR = 1.43, p = 0.04), while the overall trend lost statistical significance.

**Table 2:** Multivariable logistic regression analysis.

categories	Model 1			Model 2			Model 3		
OR (95%CI)	P-value	P for trend	OR (95%CI)	P-value	P for trend	OR (95%CI)	P-value	P for trend
AIP as continuous	1.24 (1.10-1.40)	<0.001		1.17 (1.03-1.32)	0.01		0.99(0.85-1.16)	0.95	
Quartiles^a^			<0.001			0.003			0.361
Q1(n = 244)	Ref.			Ref.			Ref.		
Q2(n = 237)	1.77(0.89-1.54)	0.23		1.13(0.85-1.49)	0.37		1.30(0.91-1.84)	0.13	
Q3(n = 258)	1.51(1.15-1.99)	0.003		1.38(1.04-1.83)	0.02		1.43(1.00-2.04)	0.04	
Q4(n = 254)	1.70(1.29-2.26)	<0.001		1.50(1.11-2.01)	0.007		1.13(0.78-1.63)	0.51	

Model 1: unadjusted

Model 2: adjusted for age, male, race, weight

Model 3: adjusted for age, male, race, weight, respiratory rate, sofa, creatinine, wbc, CHD, hypertension, CKD, vasoactive agents, ventilation

^a^AIP quartiles: Q1 (≤0.44),Q2 (0.44<AIP≤0.89),Q3 (0.89<AIP≤1.47),Q4 (SHR>1.47)

### Nonlinear associations

**Fig 2:**
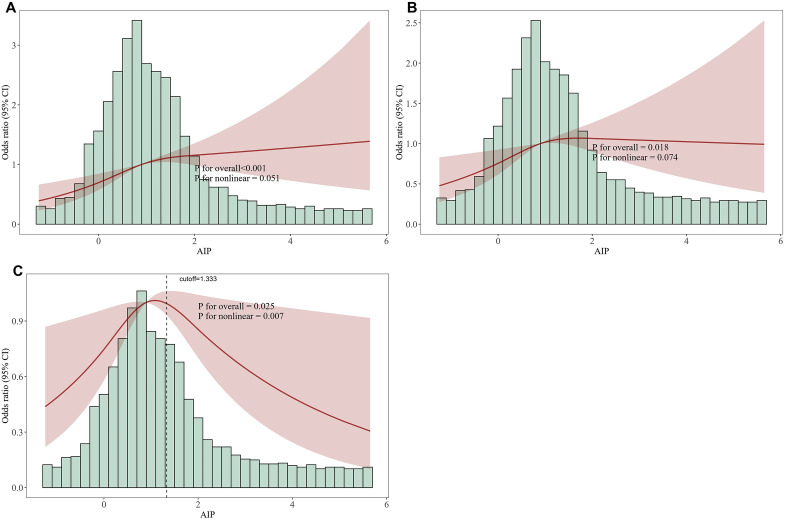
Nonlinear associations between AIP and the occurrence of AKI. **(A)** Model 1: unadjusted; **(B)** Model 2: adjusted for age, male, race, weight; **(C)** Model 3: adjusted for age, male, race, weight, respiratory rate, sofa, creatinine, wbc, CHD, hypertension, CKD, vasoactive agents, ventilation.

Fig 2 illustrates the nonlinear relationship between AIP and the risk of AKI. In the unadjusted model (Fig 2A) and the partially adjusted model (Fig 2B), no significant nonlinear association was observed. However, after full adjustment for confounding variables (Fig 2C), a significant nonlinear relationship emerged (P for nonlinearity = 0.007). The risk of AKI peaked at an AIP value of 1.333, and subsequently declined with further increases in AIP, suggesting the presence of a potential threshold effect in the association between AIP and AKI risk.

### Threshold effect analysis

As shown in **Table 3**, the standard linear regression model revealed no significant association between AIP and the risk of AKI (OR = 0.99, 95% CI: 0.85–1.16, p = 0.95). However, a two-piecewise linear regression model using 1.333 as the inflection point demonstrated a distinct threshold effect. When AIP was below 1.333, it was positively associated with AKI risk (OR = 1.329, 95% CI: 1.03–1.713, p = 0.028), whereas AIP levels above 1.333 were inversely associated with AKI risk (OR = 0.719, 95% CI: 0.551–0.948, p = 0.017). The likelihood ratio test indicated that the two-piecewise model provided a significantly better fit compared to the single linear model (p = 0.006). These findings suggest a clear threshold effect, with 1.333 identified as the turning point in the relationship between AIP and AKI risk.

**Table 3:** Threshold effect analysis of the AIP on the occurrence of AKI.

	OR (95%CI)	p
Fitting model by standard linear regression	0.99(0.85-1.16)	0.95
Fitting model by two-piecewise linear regression		
AIP < 1.333	1.329(1.03-1.713)	0.028
AIP > 1.333	0.719(0.551-0.948)	0.017
P for likelihood ratio test		0.006

The model was adjusted for age, male, race, weight, respiratory rate, sofa, creatinine, wbc, CHD, hypertension, CKD, vasoactive agents, ventilation

### Subgroup analysis

**Fig 3:**
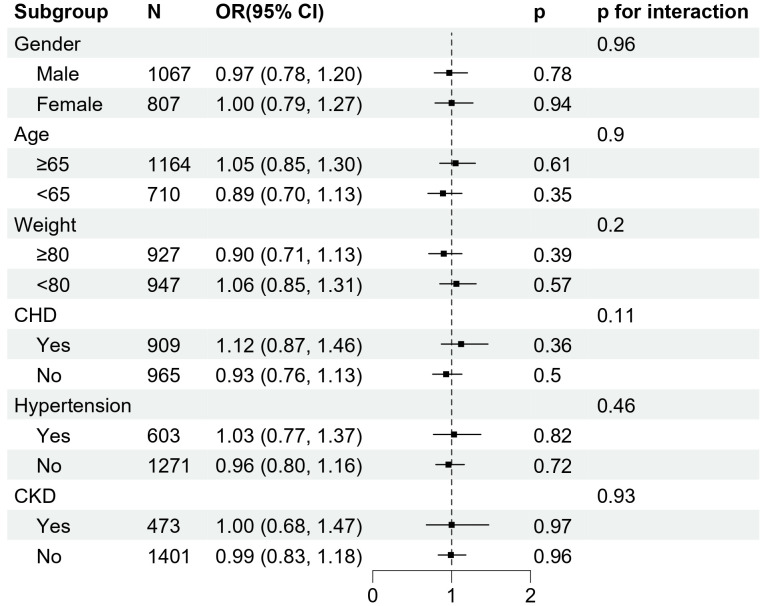
Forest plots of odds ratios for the occurrence of AKI.

Subgroup analysis revealed that the association between AIP and AKI was not statistically significant across different strata, including sex, age, body weight, CHD, CKD, and hypertension. No significant interactions were observed between AIP and any of these stratification variables (all p for interaction > 0.05), suggesting that the effect of AIP on AKI risk was consistent across various subpopulations, without evidence of effect modification by these factors (Fig 3).

**Fig 4:**
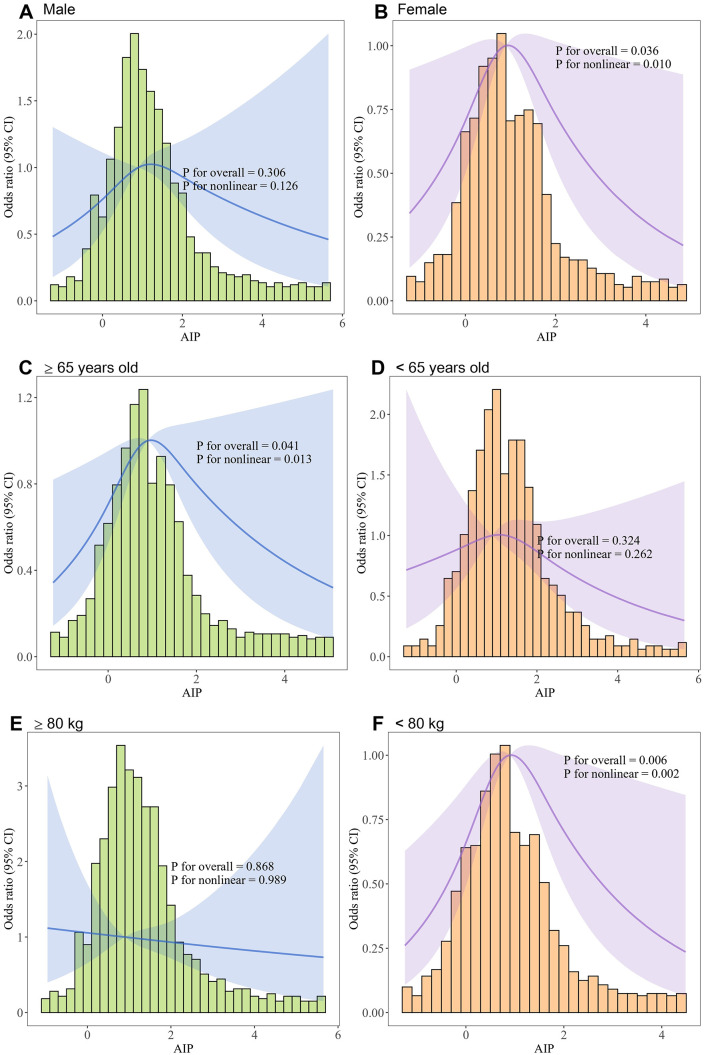
Subgroup analyses of the nonlinear association between AIP and AKI risk using restricted cubic spline models.

However, subgroup RCS analyses indicated heterogeneity in the nonlinear association between AIP and AKI risk. A significant inverted U-shaped relationship (all p for nonlinearity < 0.05) was observed in specific subgroups, including females, patients aged ≥65 years, those with body weight <80 kg, those with CHD, and patients without hypertension or CKD. In contrast, no significant nonlinear association was found in males, patients aged <65 years, those with body weight ≥80 kg, or those with hypertension or CKD. These findings suggest that the impact of AIP on AKI risk may be more pronounced in certain populations, highlighting the importance of monitoring AIP levels in these specific subgroups during clinical risk assessment (Fig 4; S1 Fig).

### Mediation analysis

**Fig 5:**
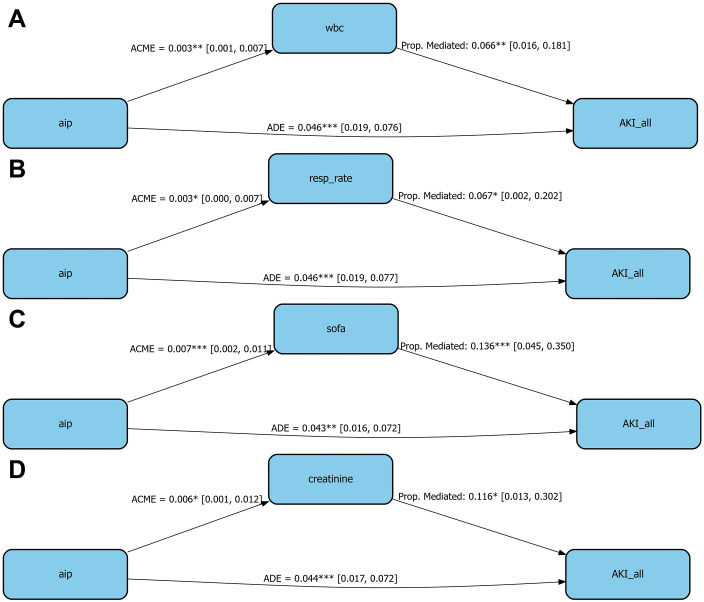
Mediation effects of clinical variables in the association between AIP and the occurrence of AKI. *means <0.05; **means <0.01; ***means <0.001. **Abbreviations:** ACME, Average Causal Mediation Effect; ADE, Average Direct Effect; Prop. Mediated, Proportion of the Effect Mediated.

Mediation analysis revealed that AIP influenced the risk of AKI indirectly through several physiological indicators. Among them, SOFA score and serum creatinine demonstrated the most pronounced mediating effects, accounting for 13.6% and 11.6% of the total effect of AIP on AKI, respectively (Fig 5). In addition, WBC count and respiratory rate also showed statistically significant mediation effects. These findings suggest that the impact of AIP on AKI risk is partially mediated through alterations in WBC, respiratory rate, SOFA score, and creatinine levels.

## Discussion

This study is the first to identify a significant nonlinear association between the AIP and AKI in patients with sepsis, further clarifying a threshold effect within this relationship. In the unadjusted and partially adjusted models, no significant nonlinear association was observed; however, after fully adjusting for confounding factors, a significant nonlinear relationship emerged, indicating that the association between AIP and AKI risk might be influenced by these potential confounders. Threshold effect analysis identified a distinct inflection point at an AIP value of 1.333, demonstrating that AIP was positively associated with AKI risk below this threshold but inversely associated above it. Additionally, subgroup analyses revealed a more pronounced nonlinear relationship within specific populations, particularly among females, patients aged ≥65 years, those weighing <80 kg, individuals with coronary heart disease, and those without hypertension or chronic kidney disease. This finding suggests that clinical attention should be particularly directed toward monitoring AIP levels in these subgroups. Lastly, mediation analysis indicated that AIP indirectly influenced the risk of AKI through several physiological parameters, including the SOFA score, serum creatinine levels, white blood cell count, and respiratory rate, partially elucidating the underlying pathophysiological mechanisms.

In the fully adjusted model (Model 3), we found that the association between AIP as a continuous variable and AKI was no longer statistically significant. This result suggests that the relationship between AIP and AKI may be significantly influenced by multiple confounding factors. Specifically, we adjusted for variables including creatinine, white blood cell count, respiratory rate, SOFA score, use of vasopressors, use of mechanical ventilation, hypertension, coronary heart disease, and chronic kidney disease. These factors are all likely to have an important impact on the relationship between AIP and AKI in septic patients. First, the effects of creatinine and CKD may contribute to the loss of statistical significance in the association between AIP and AKI. As a key indicator of kidney function, fluctuations in creatinine levels may directly influence the relationship between AIP and AKI [23]. Additionally, the underlying decline in renal function in patients with CKD could potentially alter AIP metabolism, thereby masking the relationship between AIP and AKI [24]. Second, WBC and SOFA score are markers of systemic inflammatory response. Our adjustments suggest that the intensity of the inflammatory response may play a significant role in modulating the relationship between AIP and AKI [25,26]. In particular, patients with high SOFA scores may experience changes in AIP levels due to excessive activation of the inflammatory response, which could be closely associated with the development of AKI. Finally, therapeutic interventions such as vasopressor administration and mechanical ventilation may alter hemodynamic status and oxygenation, further influencing the relationship between AIP and AKI [27,28]. Additionally, the presence of underlying cardiovascular conditions, such as hypertension and coronary heart disease, may affect the cardiac-renal axis, potentially modifying the association between AIP and AKI [29]. In conclusion, although the association between AIP and AKI is no longer significant in the fully adjusted model, we believe this does not imply the absence of a potential biological link between the two. Rather, it suggests that the relationship may be influenced by the complex interactions of these clinical variables.

Previous studies have primarily focused on mortality as the adverse outcome of interest. Significant associations have been demonstrated between AIP and both all-cause mortality and cardiovascular mortality [12,13]. Notably, these associations remain robust even in specific populations such as those with hypertension, Cardiovascular-Kidney-Metabolic syndrome, or heart failure [14,30,31]. One previous study investigated the association between AIP and the occurrence of AKI in patients with pancreatitis [18]. The results revealed a significant linear relationship between AIP and AKI risk, with no evidence of nonlinearity. In contrast, our study, which focused on critically ill patients with sepsis, identified a significant nonlinear association between AIP and the risk of AKI. The difference between our findings and those in pancreatitis patients may be attributed to variations in disease pathology and severity. Pancreatitis involves mainly localized inflammation with relatively stable metabolism, likely resulting in a linear AIP–AKI relationship. In contrast, sepsis features systemic inflammation, immune dysregulation, and multiorgan dysfunction, leading to a more complex, nonlinear association. Moderate AIP elevations may reflect metabolic activation and increased AKI risk, whereas very high levels could indicate compensatory suppression, forming an inverted U-shaped curve. We observed a threshold effect at an AIP of 1.333, where the risk of AKI decreases beyond this value. However, the biological mechanism underlying this phenomenon remains unclear. AIP is calculated as the ratio of triglycerides to HDL-C, reflecting the balance of lipid metabolism. We hypothesize that when AIP exceeds 1.333, it may indicate a compensatory change in lipid metabolism, potentially through mechanisms such as reducing inflammation, improving endothelial function, or decreasing oxidative stress, thereby lowering the risk of AKI. Although this hypothesis is biologically plausible, further experimental and clinical studies are needed to validate this mechanism.

A growing body of evidence has demonstrated a close association between insulin resistance and renal impairment. Firstly, insulin resistance-induced oxidative stress has been strongly linked to injury of glomerular endothelial cells, thickening of the glomerular basement membrane, and mesangial cell proliferation [32]. These pathological changes contribute to the development of glomerulosclerosis and tubulointerstitial damage, eventually impairing renal function. Secondly, insulin resistance can elevate catecholamine levels and disrupt the balance of pro-inflammatory cytokines and adipokines, potentially resulting in sustained hypercatecholaminemia and a chronic inflammatory state, both of which may negatively impact kidney function [33,34]. Finally, hyperinsulinemia resulting from insulin resistance may enhance renal sodium reabsorption and elevate the glomerular filtration rate, processes that over time can contribute to kidney injury [35,36]. The hyperinsulinemic-euglycemic clamp technique is widely recognized as the gold standard for evaluating IR. However, its application is limited in clinical and research settings due to its time-consuming nature, high cost, labor intensity, and requirement for specialized skills. As a result, surrogate markers like the AIP have gained attention as convenient, efficient, and dependable alternatives for assessing IR. Sepsis induces a systemic inflammatory response that disrupts normal lipid metabolism, leading to the accumulation of free fatty acids (FFAs) and triglycerides. This excess of lipids, particularly FFAs, can damage renal cells through mechanisms such as oxidative stress, mitochondrial dysfunction, and inflammation, all of which contribute to the development of AKI. The AIP, reflecting the ratio of triglycerides to HDL-C, serves as an indicator of lipid imbalance. A moderate increase in AIP signifies a state of heightened lipotoxicity, which in turn increases the risk of AKI. However, as AIP rises further, compensatory mechanisms may mitigate the negative effects of excessive lipids, such as enhanced lipid oxidation and improved HDL-C function, resulting in a reduction in AKI risk beyond a certain threshold. This may explain the threshold effect observed at an AIP of 1.333 in our study.

This study provides important insights into the mechanistic link between IR and sepsis. Understanding these underlying pathophysiological processes may facilitate the development of targeted therapeutic approaches and improve the accuracy of prognostic evaluations in the management of sepsis. Such knowledge could also support the advancement of novel treatment strategies aimed at modulating IR to improve outcomes in septic patients.

### Limitations

There are several limitations to this study. First, as a retrospective analysis based on historical data, it is susceptible to information bias. Second, due to the nature of observational research, residual confounding could not be entirely eliminated, which restricts the ability to draw causal conclusions. Third, the sample may have been affected by both recognized and unrecognized selection factors, potentially limiting the generalizability of the findings to broader populations.

## Conclusion

AIP was nonlinearly associated with AKI risk in sepsis, showing a clear threshold effect. The association was partially mediated by SOFA score, creatinine, WBC count, and respiratory rate, suggesting that AIP may influence AKI through multiple clinical pathways. These findings support the potential of AIP as a practical marker for AKI risk assessment in septic patients.

## Supporting information

S1 Fig Subgroup analyses of the nonlinear association between AIP and AKI risk using restricted cubic spline models. (TIFF)

S1 Table Univariable logistic regression analysis. (DOCX)
