# Supplementary figures and images for: Association between the atherogenic index of plasma and the occurrence of acute kidney injury in critically ill patients with sepsis: A retrospective study
Source: PLoS One. 2025 Dec 1;20(12):e0337903. doi: 10.1371/journal.pone.0337903 (PMC12668611; doi:10.1371/journal.pone.0337903)

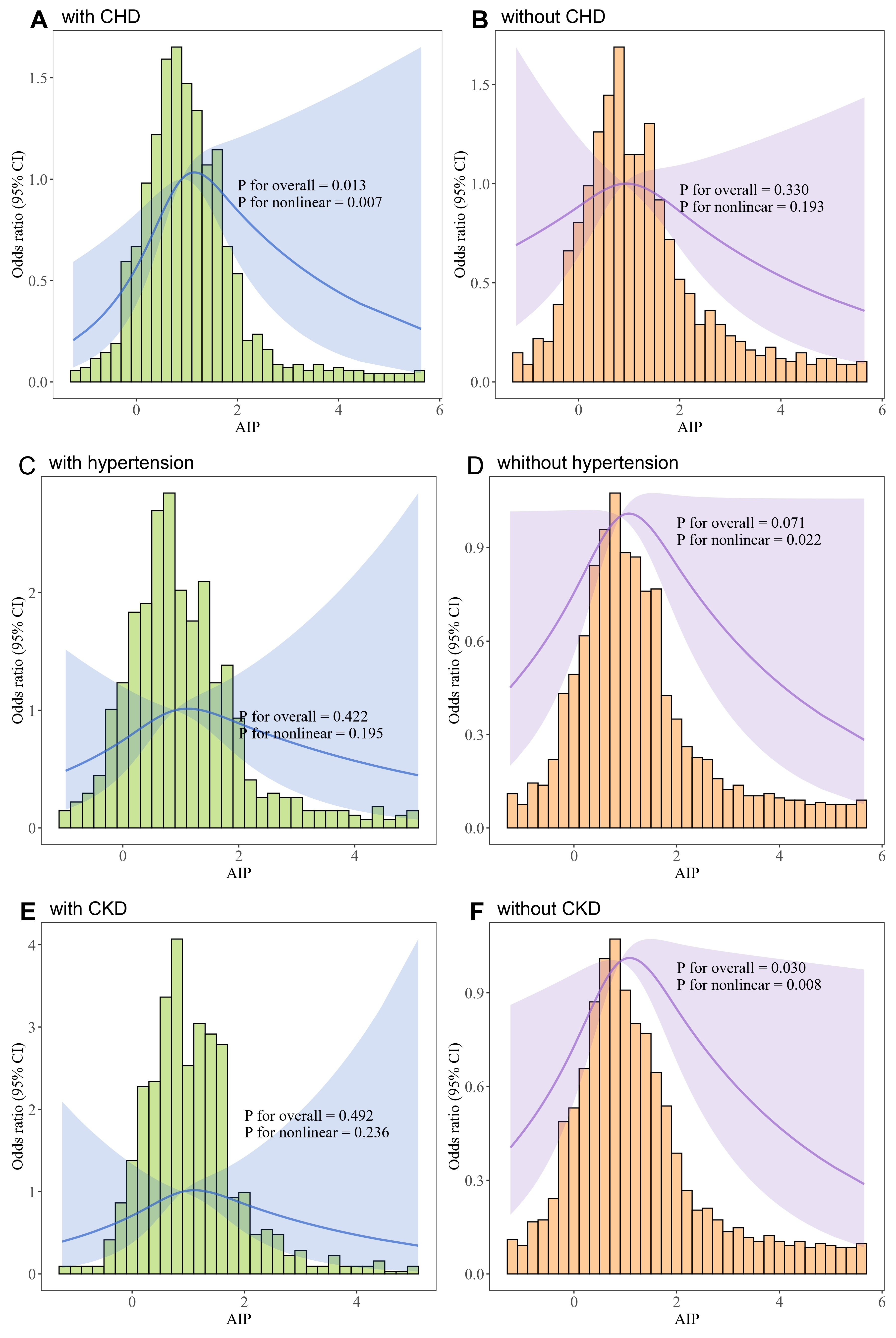

Supplement: S1 Fig — (TIFF) [file pone.0337903.s001.tiff]
