# Supplementary material for: Association between the atherogenic index of plasma and the occurrence of acute kidney injury in critically ill patients with sepsis: A retrospective study
Source: PLoS One. 2025 Dec 1;20(12):e0337903. doi: 10.1371/journal.pone.0337903 (PMC12668611; doi:10.1371/journal.pone.0337903)
Supplement: S1 Table — (DOCX) [file pone.0337903.s002.docx]

**Table S1** Univariable logistic regression analysis

| Categories | OR(95%CI) | p |
| --- | --- | --- |
| Male | 1.32(1.08-1.61) | 0.006 |
| Age | 0.99(0.98-0.99) | 0.011 |
| Race | 0.80(0.75-0.87) | <0.001 |
| Weight | 1.01(1.00-1.01) | <0.001 |
| Heart rate | 1.00(0.99-1.01) | 0.129 |
| Mean blood pressure | 0.99(0.99-1.00) | 0.446 |
| Respiratory rate | 1.02(1.00-1.04) | 0.01 |
| Spo2 | 1.01(0.98-1.04) | 0.273 |
| sofa | 1.08(1.03-1.13) | 0.001 |
| sodium | 0.99(0.97-1.01) | 0.592 |
| potassium | 1.03(0.90-1.17) | 0.641 |
| Chloride | 0.99(0.97-1.01) | 0.456 |
| creatinine | 1.14(1.04-1.25) | 0.004 |
| hemoglobin | 0.99(0.95-1.04) | 0.912 |
| BUN | 1.00(0.99-1.01) | 0.136 |
| White blood cell | 1.03(1.01-1.05) | <0.001 |
| Chronic heart disease | 0.79(0.65-0.96) | 0.021 |
| hypertension | 0.58(0.47-0.71) | <0.001 |
| Type 2 diabetes mellitus | 0.84(0.67-1.06) | 0.161 |
| Chronic kidney disease | 0.65(0.52-0.81) | <0.001 |
| vasoactive | 5.82(4.39-7.72) | <0.001 |
| ventilation | 20.55(15.61-27.05) | <0.001 |
| AIP | 1.24(1.10-1.40) | <0.001 |

Abbreviations: AIP, atherogenic index of plasma; BUN, blood urea nitrogen;


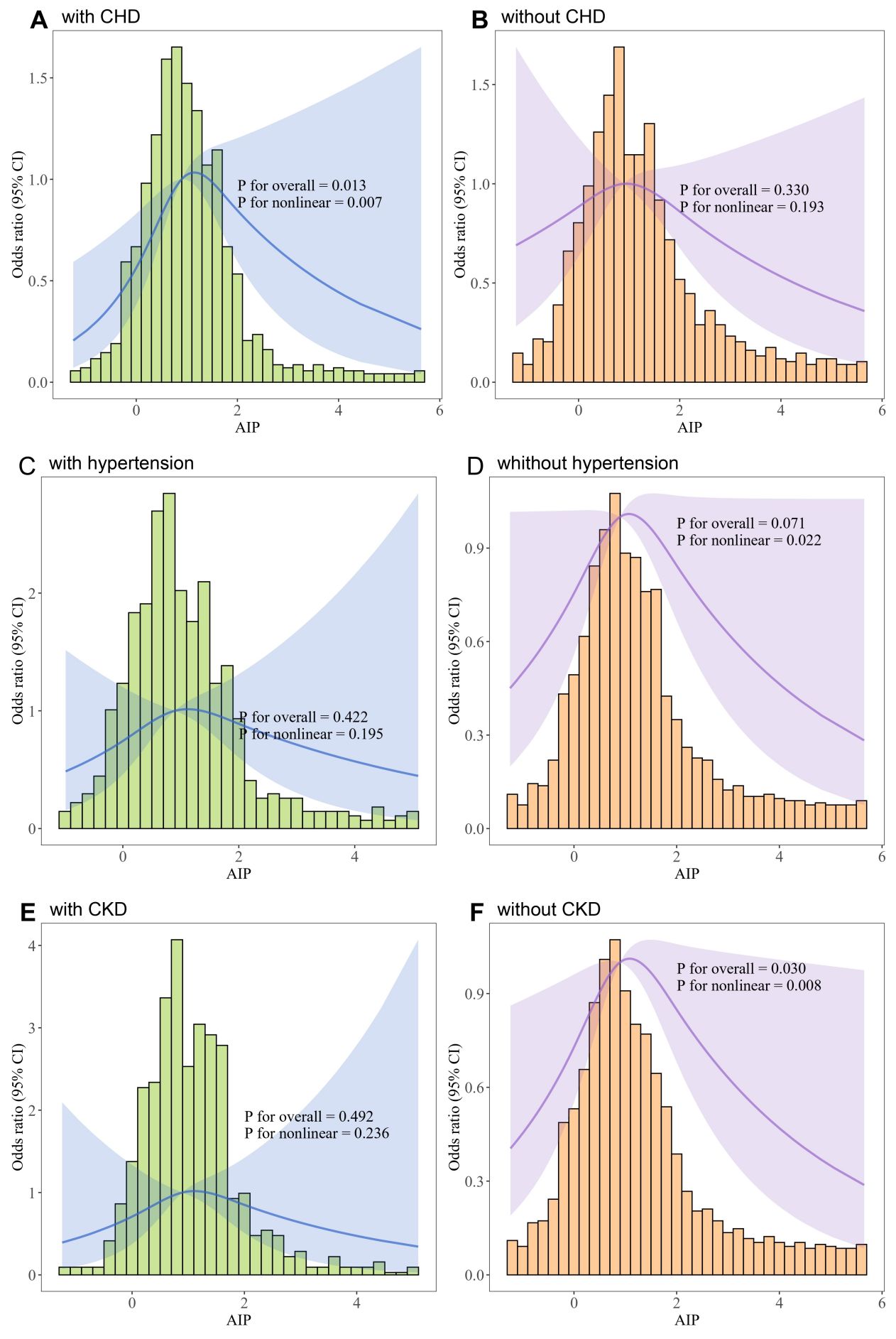


**Fig.S1** Subgroup analyses of the nonlinear association between AIP and AKI risk using restricted cubic spline models
